# *Mycobacterium leprae* genomes from naturally infected nonhuman primates

**DOI:** 10.1371/journal.pntd.0006190

**Published:** 2018-01-30

**Authors:** Tanvi P. Honap, Luz-Andrea Pfister, Genevieve Housman, Sarah Mills, Ross P. Tarara, Koichi Suzuki, Frank P. Cuozzo, Michelle L. Sauther, Michael S. Rosenberg, Anne C. Stone

**Affiliations:** 1 School of Life Sciences, Arizona State University, Tempe, Arizona, United States of America; 2 School of Human Evolution and Social Change, Arizona State University, Tempe, Arizona, United States of America; 3 California National Primate Research Center, University of California, Davis, California, United States of America; 4 Department of Clinical Laboratory Science, Teikyo University, Tokyo, Japan; 5 Lajuma Research Centre, Louis Trichardt (Machado), South Africa; 6 Department of Anthropology, University of Colorado, Boulder, Colorado, United States of America; 7 Center for Evolution and Medicine, Arizona State University, Tempe, Arizona, United States of America; University of Tennessee, UNITED STATES

## Abstract

Leprosy is caused by the bacterial pathogens *Mycobacterium leprae* and *Mycobacterium lepromatosis*. Apart from humans, animals such as nine-banded armadillos in the Americas and red squirrels in the British Isles are naturally infected with *M*. *leprae*. Natural leprosy has also been reported in certain nonhuman primates, but it is not known whether these occurrences are due to incidental infections by human *M*. *leprae* strains or by *M*. *leprae* strains specific to nonhuman primates. In this study, complete *M*. *leprae* genomes from three naturally infected nonhuman primates (a chimpanzee from Sierra Leone, a sooty mangabey from West Africa, and a cynomolgus macaque from The Philippines) were sequenced. Phylogenetic analyses showed that the cynomolgus macaque *M*. *leprae* strain is most closely related to a human *M*. *leprae* strain from New Caledonia, whereas the chimpanzee and sooty mangabey *M*. *leprae* strains belong to a human *M*. *leprae* lineage commonly found in West Africa. Additionally, samples from ring-tailed lemurs from the Bezà Mahafaly Special Reserve, Madagascar, and chimpanzees from Ngogo, Kibale National Park, Uganda, were screened using quantitative PCR assays, to assess the prevalence of *M*. *leprae* in wild nonhuman primates. However, these samples did not show evidence of *M*. *leprae* infection. Overall, this study adds genomic data for nonhuman primate *M*. *leprae* strains to the existing *M*. *leprae* literature and finds that this pathogen can be transmitted from humans to nonhuman primates as well as between nonhuman primate species. While the prevalence of natural leprosy in nonhuman primates is likely low, nevertheless, future studies should continue to explore the prevalence of leprosy-causing pathogens in the wild.

## Author summary

*Mycobacterium leprae*, which causes leprosy in humans, also infects nine-banded armadillos, red squirrels, and nonhuman primates. Genomic data for *M*. *leprae* strains from wild armadillos and red squirrels show that humans were responsible for the original introduction of *M*. *leprae* to these species. It is not known whether naturally occurring leprosy among nonhuman primates is due to incidental infections from humans or whether nonhuman primates can serve as a host for *M*. *leprae*. To this end, we sequenced complete genomes of *M*. *leprae* strains from three naturally infected nonhuman primates. Our results suggest that *M*. *leprae* strains can be transmitted from humans to nonhuman primates as well as between nonhuman primate species, and thus, other primates might serve as a host for *M*. *leprae* in the wild. We also assessed whether wild ring-tailed lemurs from Madagascar and chimpanzees from Uganda showed presence of *M*. *leprae* infection. Although these populations tested negative for *M*. *leprae* infection, further research on the prevalence of *M*. *leprae* in other wild nonhuman primate populations, especially in leprosy-endemic regions, is warranted.

## Introduction

Leprosy has afflicted mankind for many millennia and remains a highly prevalent disease in economically underprivileged countries. Due to effective multi-drug therapy, the global prevalence of leprosy has been reduced to less than one case per 10,000 individuals [1]. The disease has been almost eradicated from developed countries; however, approximately 250,000 new leprosy cases occur each year worldwide [1]. The majority of these cases occur in tropical and subtropical countries including India, Brazil, and the Central African Republic, thereby making leprosy a Neglected Tropical Disease [1].

Leprosy mainly affects the skin, mucosa of the nose and upper respiratory tract, and the peripheral nervous system. Depending on the host immune response, the infection can progress to either the tuberculoid (paucibacillary) or lepromatous (multibacillary) form of leprosy. Tuberculoid leprosy is characterized by the presence of one or a few hypopigmented patches with loss of sensation and thickened peripheral nerves, whereas lepromatous leprosy results in systemic lesions which may become infiltrated with fluids [2]. If left untreated, permanent nerve damage can occur, and secondary infections can lead to tissue loss resulting in disfigurement of the extremities [2]. The pathogen has a long incubation period that averages three to five years and can extend up to thirty years, which hampers early detection of infection.

In humans, leprosy is caused by the bacterial pathogens, *Mycobacterium leprae* and *Mycobacterium lepromatosis*, the latter of which also causes diffuse lepromatous leprosy [3,4]. While *M*. *leprae* causes the majority of leprosy cases and is prevalent worldwide, *M*. *lepromatosis* is mainly endemic to Mexico and the Caribbean [5–7], although isolated cases have been reported from other countries [8,9]. *M*. *leprae* and *M*. *lepromatosis* show approximately 88% genetic identity and are estimated to have diverged 13–14 million years ago (MYA) [10]. Despite this deep divergence, they share many common characteristics such as a reduced overall genome size (relative to other mycobacteria) of approximately 3.2 million base pairs (bp), similar genome organization, and the inability to grow outside of a living host. This obligate intracellular parasitism is the result of a reductive evolution event that occurred about 12–20 MYA in the genome of the common ancestor of *M*. *leprae* and *M*. *lepromatosis* leading to the loss of functionality of a number of genes in both species [10,11].

The lack of paleopathological evidence of leprosy in the pre-contact era Americas [12] as well as genetic data showing that *M*. *leprae* strains currently circulating in the Americas are closely related to medieval European *M*. *leprae* strains [13–15] suggest that leprosy was brought to the Americas by European settlers. Traditionally thought to be an exclusively human pathogen, *M*. *leprae* has been found to infect other animals. For example, nine-banded armadillos in the Americas were naturally infected with *M*. *leprae* long before their use as laboratory models [16] and therefore, must have originally acquired the pathogen from infected humans. How this occurred is unknown, but ingestion of human garbage has resulted in transmission of other mycobacteria, specifically *Mycobacterium tuberculosis*, to nonhuman primates [17,18]. Recently, red squirrel populations in the UK were found to carry *M*. *leprae* as well as *M*. *lepromatosis* [19]. The red squirrel *M*. *leprae* strains belong to the same *M*. *leprae* lineage as that recovered from medieval European humans [19], and hence, it is likely that the original introduction of *M*. *leprae* to red squirrels occurred centuries earlier when leprosy was still prevalent in the region. While the mechanisms of transmission from animals such as armadillos or red squirrels to humans are unclear, the most likely route is aerosol transmission during extended contact. For example, the squirrel fur trade could have played a role in the transmission of leprosy between red squirrels and humans [19,20]. Today, in the southeastern US, leprosy cases have been reported in US-born individuals with no prior residence in a foreign country and no known contact with leprosy patients. Interestingly, many of these patients are infected with a genotype of *M*. *leprae* that is not currently circulating in human populations in other parts of the world, but is prevalent in wild armadillos in these states [21,22], suggesting zoonotic transmission of *M*. *leprae* from armadillos to humans in these regions. Close contact with armadillos as well as processing and/or consumption of infected armadillo meat may be mechanisms for transmission of leprosy between armadillos and humans [22].

Nonhuman primates including white-handed gibbons, rhesus macaques, African green monkeys, sooty mangabeys, and chimpanzees are capable of being experimentally infected with *M*. *leprae* resulting in symptomatic leprosy similar to that observed in humans [23]. Furthermore, isolated cases of naturally occurring leprosy have been observed in nonhuman primates such as chimpanzees [24–28], sooty mangabeys [29,30], and cynomolgus macaques [31]. In these cases, the nonhuman primates were captured from the wild and imported to research facilities for experimental purposes. The animals were not experimentally infected with *M*. *leprae* nor did they have close contact with a known leprosy patient. All animals developed symptoms characteristic of human leprosy, and in most cases, the etiological agent was confirmed to be *M*. *leprae* using microscopic or genetic analyses. However, the genomes of these nonhuman primate *M*. *leprae* strains have not been previously sequenced. In this study, we sequenced *M*. *leprae* genomes from three naturally infected nonhuman primates–a chimpanzee from Sierra Leone [28], a sooty mangabey from West Africa [29], and a cynomolgus macaque from The Philippines [31]. The details of these three cases are given in S1 Table.

Additionally, this study aimed to assess whether *M*. *leprae* and other mycobacterial pathogens are prevalent in wild nonhuman primates living in contact with human populations. We screened ring-tailed lemurs from the Bezà Mahafaly Special Reserve (BMSR), Madagascar, and chimpanzees from Ngogo, Kibale National Park, Uganda, for the presence of mycobacterial infection using quantitative PCR (qPCR) assays.

## Methods

### Sequencing the genomes of nonhuman primate *M*. *leprae* strains

#### Sampling

Firstly, we acquired a sample of *M*. *leprae* DNA previously extracted from a naturally infected sooty mangabey (*Cercocebus atys*) [29]. The *M*. *leprae* strain was isolated by passaging in an armadillo that had tested negative for naturally acquired *M*. *leprae* infection [29] and bacterial DNA was extracted using the protocol given in [32]. Secondly, we acquired a sample of DNA previously extracted from the skin biopsy of a naturally infected female chimpanzee (*Pan troglodytes verus*) [28]. Lastly, we acquired a sample of skin biopsy tissue from a naturally infected cynomolgus macaque (*Macaca fascicularis*). The skin biopsy sample had been stored using the formalin-fixed paraffin-embedded (FFPE) method since 1994 [31].

#### Ethics statement

The cynomolgus macaque was maintained at the California National Primate Research Center, University of California, Davis, in accordance with established standards of the U.S. Federal Animal Welfare Act, the American Association for Accreditation of Laboratory Animal Care (AAALAC), and the Guide for the Care and Use of Laboratory Animals [33], as given in [31]. The animal was originally acquired under a Convention on International Trade in Endangered Species of Wild Fauna and Flora (CITES) export permit # 4455. A CITES permit was not required for the *M*. *leprae* DNA samples which had been previously extracted from the chimpanzee and sooty mangabey. Hereafter, the chimpanzee, sooty mangabey, and cynomolgus macaque samples will be referred to as Ch4, SM1, and CM1, respectively.

#### DNA extraction

We extracted DNA from sample CM1 using the DNeasy Blood and Tissue Kit (Qiagen). 0.5 g of tissue was used as starting material and the extraction was carried out using the manufacturer’s protocol with the following modification: DNA was eluted in 100 μL AE buffer (Qiagen) that had been preheated to 65°C. A qPCR assay targeting the *M*. *leprae*-specific multi-copy *rlep* element [34] was used to confirm the presence of *M*. *leprae* DNA.

#### *M*. *leprae* genome sequencing

The SM1 *M*. *leprae* DNA sample was converted into a paired-end fragment library using the GS FLX Titanium General Library Preparation Kit (Roche) and the manufacturer’s protocol. The library was sequenced using the 454 GS-FLX Titanium sequencer (½ 70 × 75 PicoTiterPlate GS XLR70 run) at SeqWright DNA Technology Services, Texas, US.

The Ch4 and CM1 DNA extracts were sheared to an average size of 300 bp using the M220 Focused-ultrasonicator (Covaris) and converted into double-indexed DNA libraries using a library preparation protocol based on [35]. For sample CM1, two separate libraries were prepared (namely, CM1_Lib1 and CM1_Lib2). Libraries were quantified using the Bioanalyzer 2100 DNA1000 assay (Agilent) and the KAPA Library Quantification kit (Kapa Biosystems). The libraries were target enriched for the *M*. *leprae* genome using a custom MYbaits Whole Genome Enrichment kit (MYcroarray). Specifically, biotinylated RNA baits were prepared using DNA from *M*. *leprae* Br4923, Thai53, and NHDP strains. 57 ng of the CH4 library, 467 ng of CM1_Lib1, and 910 ng of CM1_Lib2 were used for enrichment. Each library was enriched in a separate reaction. Enrichment was conducted according to the MYbaits protocol with hybridization being carried out at 65°C for 24 hours. After elution, the CH4 library and the CM1_Lib1 were amplified using AccuPrime *Pfx* DNA polymerase (Life Technologies) for 27 and 23 cycles, respectively, following the protocol given in [36]. The enriched CM1_Lib2 was amplified over two separate reactions, each for 14 cycles, using KAPA HiFi polymerase (Kapa Biosystems). All amplification reactions were cleaned up using the MinElute PCR Purification kit (Qiagen). Two library blank samples (PCR-grade water) were also processed into libraries and target-enriched in a similar manner to ensure that no contamination had been introduced during the process; these are referred to as LB1 and LB2. All samples (Ch4, CM1_Lib1, CM1_Lib2, LB1, and LB2) were sequenced over two sequencing runs on the Illumina HiSeq 2500 using the Rapid PE v2 chemistry (2 ×100 bp) at the Yale Center for Genome Analysis, Connecticut, US. These runs also included samples from other ongoing research projects; however, firstly, none of these samples contained mycobacterial DNA, and secondly, only reads containing the appropriate combination of unique indices were used for data analyses, therefore, the chances of cross-contamination were negligible.

#### Data processing and mapping

For sample SM1, the FASTA and QUAL files obtained from the sequencing facility were combined into a FASTQ file using the Combine FASTA and QUAL tool on the Galaxy server (https://usegalaxy.org). Reads were trimmed using AdapterRemoval v2 with default parameters [37]. For samples Ch4 and CM1, paired-end reads were trimmed and merged using SeqPrep (https://github.com/jstjohn/SeqPrep) with the following modification: the minimum overlap for merging was set to 11. Since sample CM1 had two separately sequenced libraries, paired-end reads for each library were trimmed and merged separately, and the merged reads were concatenated.

For all samples including the library blanks, reads were mapped to the *M*. *leprae* TN reference genome (AL450380.1) using the Burrows Wheeler Aligner (bwa) v0.7.5 [38] with default parameters. SAMtools v0.1.19 [39] was used to filter the mapped reads for a minimum Phred quality threshold of Q37 and remove PCR duplicates and reads with multiple mappings.

To determine the percentage of reads mapping to the host genome, reads for samples Ch4, SM1, and CM1 were also mapped to the *Pan troglodytes* reference genome (GCA_000001515.4), the *Dasypus novemcinctus* reference genome (GCA_000208655.2), and the *Macaca fascicularis* reference genome (GCA_000364345.1), respectively, using similar methodology as given above.

#### Comparative data

Publicly-available Illumina reads for five ancient (Jorgen625, Refshale16, SK2, SK8, and 3077) and eight modern (S2, S9, S10, S11, S13, S14, S15, and Airaku3) human *M*. *leprae* strains and the Brw15-20m strain (representative of the red squirrel *M*. *leprae* clade) were acquired from the Sequence Read Archive. Reads were processed and mapped to the *M*. *leprae* TN reference genome using the same methodology as described above. FASTA files for the finished *M*. *leprae* genomes (Br4923, Kyoto2, NHDP63, and Thai53) were acquired from GenBank and aligned to the *M*. *leprae* TN reference genome using LAST with default parameters [40]. Similarly, contigs for *M*. *lepromatosis* Mx1-22A (JRPY00000000.1) were acquired from GenBank and aligned to the *M*. *leprae* TN reference genome using LAST with the gamma-centroid option as given in [10]. The maf-convert option was used to convert the alignment files to SAM files, and SAMtools was used obtain BAM files which were used for further analyses.

#### Variant calling

For the BAM files obtained after processing genomes from Illumina dataset, an mpileup file was generated using SAMtools and processed using VarScan v2.3.9 [41]. A VCF file containing all sites (variant as well as invariant) was produced using the following parameters: minimum number of reads covering the position = 5, minimum number of reads covering the variant allele = 3, minimum variant frequency = 0.2, minimum base quality = 30, and maximum frequency of reads on one strand = 90%. For the finished *M*. *leprae* genomes and *M*. *lepromatosis*, SAMtools (v1.3.1) mpileup and bcftools call were used to produce the VCF files. VCF files for all strains were combined using the CombineVariants tool available in the Genome Analysis Toolkit (GATK) [42]. VCFtools [43] was used to remove insertions and deletions and exclude positions which occurred in known repeat regions and rRNA and positions covered by the SK12 negative control sample [15]. The list of all positions excluded from the analyses is given in S1 File. The SelectVariants tool in GATK was used to output a VCF file containing only the single nucleotide polymorphisms (SNPs). Positions where one or more strains had an unknown or missing nucleotide were excluded. SNP calls were manually checked for possible errors or inconsistencies. A publicly available perl script [44] was used to generate an alignment comprising those positions where at least one of the strains had a SNP.

#### Phylogenetic analyses

Phylogenetic trees were constructed using the Neighbor-Joining (NJ) and Maximum Parsimony (MP) methods in MEGA7 [45] as well as using a Bayesian approach in BEAST v1.8.4 [46]. The SNP alignment of all the *M*. *leprae* genomes and *M*. *lepromatosis* comprised 233,509 sites and was used as input for MEGA7. The NJ tree was generated using the *p*-distance method. This method was used because the alignment did not contain invariant sites and the *M*. *leprae* genomes are not highly diverged. Bootstrap support was estimated from 1,000 replicates. The MP tree was generated using the Subtree-Pruning-Regrafting (SPR) algorithm and 1,000 bootstrap replicates.

**Fig 1:**
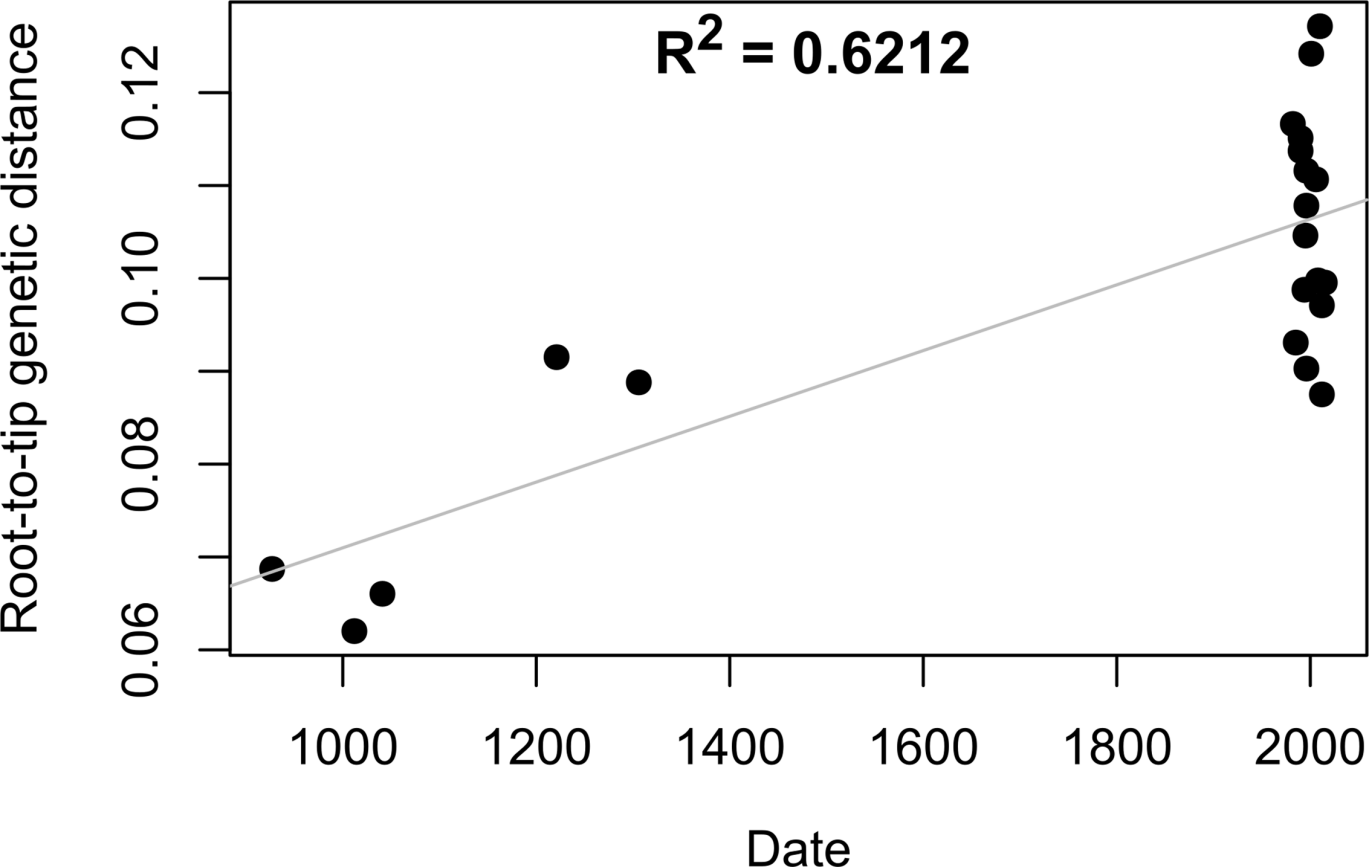
Scatter plot of date vs genetic distance of *M*. *leprae* strains. The x-axis denotes mean date in CE (calibrated radiocarbon date for ancient strains and isolation year for modern strains). The y-axis denotes root-to-tip genetic distance for each strain.

To determine divergence times of the *M*. *leprae* strains, a SNP alignment of only the *M*. *leprae* strains was generated. Sites with missing or unknown data were removed, resulting in an alignment comprising 747 sites. The modern human *M*. *leprae* strain S15 was excluded from this analysis because it contains an unusually high number of SNPs, likely related to its multi-drug resistance [15]. To assess whether there was a sufficient temporal signal in the data to proceed with molecular clock analysis, a regression of root-to-tip genetic distance against dates of the *M*. *leprae* strains was conducted using TempEst [47]. The R^2^ value calculated in TempEst equaled 0.6212, signifying a positive correlation between genetic divergence and time for the *M*. *leprae* strains (Fig 1). Therefore, the data were found to be suitable for molecular clock analysis.

The SNP alignment was analyzed using BEAST v1.8.4 [46]. The calibrated radiocarbon dates of the ancient strains in years before present (YBP, with present being considered as 2017), the isolation years of the modern strains, and a substitution rate of 6.87 × 10^−9^ substitutions per site per year as estimated by [19] were used as priors. Using jModelTest2 [48], the Kimura 3-parameter model with unequal base frequencies was determined to be the best model of nucleotide substitution. A strict clock model with uniform rate across branches and a tree model of constant population size were used. To account for ascertainment bias that might result from using only variable sites in the alignment, the number of invariant sites (number of constant As, Cs, Ts, and Gs) was included in the analysis. One Markov Chain Monte Carlo (MCMC) run was carried out with 50,000,000 iterations, sampling every 2,000 steps. The first 5,000,000 iterations were discarded as burn-in. Tracer [49] was used to visualize the results of the MCMC run. TreeAnnotator [46] was used to summarize the information from the sample of trees produced onto a single target tree calculated by BEAST, with the first 2,500 trees being discarded as burn-in. Figtree (http://tree.bio.ed.ac.uk/software/figtree/) was used to visualize the Maximum Clade Credibility (MCC) tree.

#### SNP analysis

The VCF files for the Ch4, SM1, and CM1 samples were analyzed using snpEff v4.3 [50]. The program was run using default parameters, except the parameter for reporting SNPs that are located upstream or downstream of protein-coding genes was set to 100 bases.

### Screening wild nonhuman primates for presence of mycobacterial pathogens

#### Sampling

Buccal swab samples were collected from wild ring-tailed lemurs, *Lemur catta*, (n = 41) from BMSR, Madagascar, in the 2009 field season. Fruit wadge samples were collected from wild chimpanzees, *Pan troglodytes schweinfurthii*, (n = 22) from Ngogo, Kibale National Park, in the 2010 field season.

#### Ethics statement

Sampling was conducted according to the American Society of Primatologists’ Principles for the Ethical Treatment of Non-Human Primates and received IACUC approval (University of North Dakota IACUC Protocol #0802–2 and animal assurance number A3917-01), as well as being permitted by the Convention on International Trade in Endangered Species (CITES Madagascar: 531C-EA10/MG10; CITES US: 11US040035/9) and Madagascar National Parks (086/12/MEF/SG/DGF/DCB.SAP/SCB). A CITES export permit was not required for the chimpanzee fruit wadge samples.

#### DNA extractions

DNA was extracted from the buccal swab samples using a phenol-chloroform DNA extraction protocol [51] and from the fruit wadge samples using the DNeasy Plant Maxi Kit (Qiagen) following the manufacturer’s instructions. For each batch of DNA extractions, a negative control sample (extraction blank) was kept to ensure that no contamination was introduced during the DNA extraction process.

#### qPCR assays

All extracts as well as extraction blanks were tested for the presence of *M*. *leprae* DNA using two TaqMan qPCR assays–one targeting the multi-copy *rlep* repeat element [34] and another targeting the single-copy *fbpB* gene, which codes for the antigen 85B [52]. Similarly, all extracts were also tested using qPCR assays targeting the mycobacterial single-copy *rpoB* gene, which codes for RNA polymerase subunit B [53], and the multi-copy insertion element IS6110, which is found in most *Mycobacterium tuberculosis* complex (MTBC) strains [54,55]. The *rpoB* assay used in this study targets members of the MTBC as well as some closely related mycobacteria such as *M*. *marinum*, *M*. *avium*, *M*. *leprae*, *M*. *kansasii*, and *M*. *lufu* [53]. The sequences of the qPCR primers and probes used for these assays are given in [56]. DNA from *M*. *leprae* SM1 and *M*. *tuberculosis* H37Rv strains were used to create DNA standards for the appropriate qPCR assays. Ten-fold serial dilutions ranging from one to 100,000 copy numbers of the genome per μL were used to plot a standard curve for quantification purposes. Non-template controls (PCR-grade water) were also included on each qPCR plate. The DNA extracts, extraction blanks, and non-template control were run in triplicate whereas DNA standards were run in duplicate for each qPCR assay. qPCR reactions were run in a 20 μL total volume: 10 μL of TaqMan 2X Universal MasterMix (Applied Biosystems), 0.2 μL of 10mg/mL RSA (Sigma), and 2 μL of sample (DNA, standard, or non-template control). Primers and probe were added at optimized concentrations as given in [53,56]. The qPCR assays were carried out on the Applied Biosystems 7900HT thermocycler with the following conditions: 50°C for 2 minutes, 95°C for 10 minutes, and 50 cycles of amplification at 95°C for 15 seconds and 60°C for 1 minute. The results were visualized using SDS 2.3 (Applied Biosystems). Both amplification and multicomponent plots were used to classify the replicates of the extracts as positive or negative. An extract was considered positive for a qPCR assay if two or more replicates out of three were positive.

## Results

### Sequencing the genomes of the nonhuman primate *M*. *leprae* strains

#### Post-mapping analysis

A total of 97–98% of the *M*. *leprae* genome was recovered for samples Ch4, SM1, and CM1 with mean coverage ranging from 13- to 106-fold (Table 1). For the library blank samples, LB1 and LB2, only ~6% of processed reads mapped to the *M*. *leprae* TN genome resulting in less than 0.1% of the *M*. *leprae* genome being covered. For sample SM1, which was shotgun-sequenced, only 2.2% of processed reads mapped to the host (armadillo) genome. For samples Ch4 and CM1, which were enriched for the *M*. *leprae* genome prior to sequencing, 16.4% of Ch4 processed reads mapped to the chimpanzee genome, whereas 52.4% of CM1 processed reads mapped to the cynomolgus macaque genome, signifying that the *M*. *leprae* capture was more effective for sample Ch4.

**Table 1:** Results of whole-genome sequencing of nonhuman primate *M*. *leprae* strains.

Strain	Host species	Raw Reads	Processed Reads ^a^	Mapped reads	Analysis-ready reads ^b^	Average read length	Mean fold-coverage	Percent genome covered ≥ one-fold
Ch4	Chimpanzee	55,710,090	50,164,345	41,193,171	3,463,490	100.7	106.8	98.0
SM1	Sooty mangabey	697,450	526,512	349,276	293,217	279.8	25.1	98.8
CM1	Cynomolgus macaque	Lib1: 17,065,716 Lib2: 32,883,154	Lib1: 14,101,593 Lib2: 30,595,430 Total: 44,697,023	12,158,918	541,153	80.2	13.3	97.7

^a^ Reads used as input for mapping after adapter trimming, merging, and removing reads less than 30 bp in length.

^b^ Reads after filtering at Q37 quality threshold, removing duplicates, and removing reads with multiple mappings

#### Phylogenetic analyses

Trees constructed using MP (S1 Fig) and NJ (S2 Fig) methods supported identical topologies for the *M*. *leprae* phylogeny. The Ch4 and the SM1 strains belong to *M*. *leprae* Branch 4. Within Branch 4, the Ch4 and SM1 strains are closely related to each other and form their own sublineage. On the other hand, the CM1 strain belongs to *M*. *leprae* Branch 0 and is most closely related to the modern human *M*. *leprae* strain S9 from New Caledonia.

**Fig 2:**
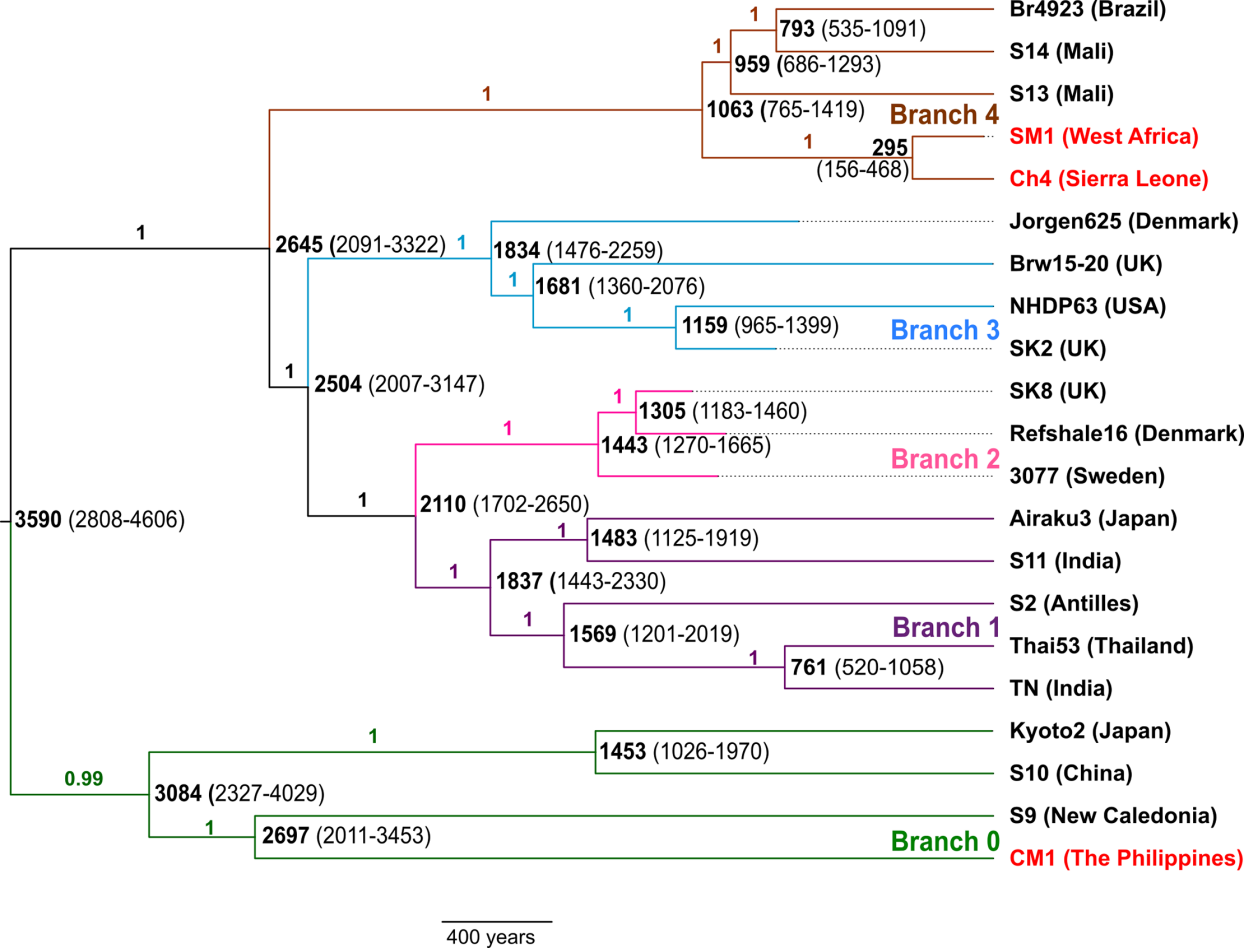
Maximum clade credibility tree of *M*. *leprae* strains. The five *M*. *leprae* branches are highlighted. Nodes are labeled with median divergence times in years before present, with the 95% HPD given in brackets. Posterior probabilities for each branch are shown next to the branches. The nonhuman primate *M*. *leprae* genomes sequenced in this study are marked in red.

According to the MCC tree (Fig 2), the Ch4 and SM1 strains diverged 295 YBP with a 95% Highest Posterior Density (HPD) range of 156–468 YBP. The sublineage comprising these two strains last shared a common ancestor with the Branch 4 human *M*. *leprae* strains 1,063 YBP (95% HPD 765–1,419 YBP). On the other hand, the CM1 strain shows a very deep divergence time of 2,697 YBP (95% HPD 2,011–3,453 YBP) from its closest relative, *M*. *leprae* strain S9. Lastly, the most recent common ancestor (MRCA) of all *M*. *leprae* strains was estimated to have existed 3,590 YBP (95% HPD 2,808–4,606 YBP). The *M*. *leprae* substitution rate was estimated to be 6.95 × 10^−9^ substitutions per site per year.

#### SNP-effect analysis

The Ch4, SM1, and CM1 strains showed 129, 124, and 167 total SNPs, respectively. A list of SNPs found in the nonhuman primate *M*. *leprae* strains and their effects are given in S2 Table. 18 SNPs were found to be unique to the Ch4-SM1 sublineage (i.e. they have so far not been found in any of the human *M*. *leprae* genomes). Additionally, the Ch4, SM1, and CM1 strains showed 9, 4, and 54 unique SNPs, respectively. A summary of the SNP-effect analysis is given in Table 2.

**Table 2:** Summary of SNP-effect analysis for the nonhuman primate *M*. *leprae* strains.

Type of variant	Ch4	SM1	CM1
missense variant in protein-coding gene	36 (4)	34 (2)	54 (20)
start loss variant in protein-coding gene	1 (0)	1 (0)	1 (0)
synonymous variant in protein-coding gene	24 (1)	24 (1)	28 (8)
variant in pseudogene	45 (3)	42 (0)	51 (10)
variant in intergenic region	23 (1)	23 (1)	33 (16)
**Total**	**129 (9)**	**124 (4)**	**167 (54)**

Values represent total number of variants in strain (number of variants unique to the strain)

### Screening of wild nonhuman primates for presence of mycobacterial pathogens

#### qPCR screening

All ring-tailed lemur and chimpanzee samples tested negative for *M*. *leprae* DNA based on the *rlep* and 85B qPCR assays. All samples also tested negative for the *rpoB* and IS6110 qPCR assays signifying the absence of infection by pathogens belonging to the MTBC.

## Discussion

### Sequencing the genomes of the nonhuman primate *M*. *leprae* strains

The Ch4 and SM1 *M*. *leprae* strains belong to *M*. *leprae* Branch 4. Human *M*. *leprae* strains belonging to this branch have been found in populations in West Africa and the Caribbean. The presence of Branch 4 strains in the Caribbean is likely due to the movement of people from Africa to the Caribbean during the slave trade [14]. Strain S15, which was isolated from a human patient from New Caledonia, also falls in Branch 4.

The Ch4 *M*. *leprae* strain was isolated from a female chimpanzee captured from Sierra Leone in 1980 and held at a research facility in Japan. The chimpanzee developed symptoms of leprosy in 2009 [28]. Since the Ch4 strain is West African in origin, the chimpanzee was likely infected in Sierra Leone before being sent to Japan. The SM1 *M*. *leprae* strain was isolated from a West African sooty mangabey (originally denoted as individual A015). This mangabey was shipped from Nigeria to the US in 1975 and developed symptoms of leprosy in 1979. It is the first of two known cases of naturally occurring leprosy in sooty mangabeys [29]. The second sooty mangabey is thought to have acquired leprosy from A015 while both animals were housed together in the US [30]. The *M*. *leprae* strain isolated from A015 was reported to be partially resistant to dapsone [29], suggesting the sooty mangabey might have acquired leprosy directly or indirectly from a human patient who had received dapsone treatment. Mutations in the *folP1* gene, for example, the Thr^53^Ile and Pro^55^Leu substitutions, are known to be associated with dapsone-resistance [57]. However, we did not find any mutations in the *folP1* gene of the SM1 strain. Therefore, the previously reported partial resistance to dapsone might be due to laboratory error or mutations in other genes which have not yet been clinically proven to cause dapsone-resistance.

A total of 18 SNPs were found to be unique to the Ch4-SM1 sublineage. These included seven missense variants occurring in genes coding for proteasome-related factors, glutamine-dependent NAD synthetase, acetyltransferases, and integral membrane proteins. The close relationship of the Ch4 and SM1 strains suggests that *M*. *leprae* might be transmitted between chimpanzees and sooty mangabeys in the wilds of Africa. The geographic range of chimpanzees overlaps with that of sooty mangabeys (S3 Fig). Chimpanzees are also known to hunt and kill other primates including mangabeys [58,59] and can acquire pathogens during predation and via consumption of infected bushmeat [60]. Since *M*. *leprae* can be transmitted through consumption of infected animal meat, this might be one of the possible routes for transmission of *M*. *leprae* among species of nonhuman primates.

The CM1 strain belongs to *M*. *leprae* Branch 0. This branch also includes strains from New Caledonia, Japan and China, and is the most deeply diverged branch of the *M*. *leprae* phylogeny [15]. The CM1 strain has 167 SNPs, out of which 54 have so far not been found in other *M*. *leprae* strains. Interestingly, the CM1 strain includes 54 missense variants, of which five variants occurred in genes belonging to the ESX system. The ESX system is a Type VII secretion system that comprises proteins which help pathogens resist or evade the host immune response [61]. The CM1 strain showed three variants in the *ML0049* gene including a unique Ala^87^Thr substitution, as well as a unique Glu^273^Lys substitution in the *ML0054* gene. The *ML0049* and *ML0054* genes belong to the ESX-1 gene system, which encodes proteins that are major determinants of virulence in *M*. *leprae*, *M*. *tuberculosis*, *M*. *kansasii*, and *M*. *marinum* [61]. They help the pathogen escape from the phagosome, thereby allowing further replication, cytolysis, necrosis, and intercellular spread [62].

The CM1 strain was recovered from a cynomolgus macaque that was shipped to the US from The Philippines in 1990. The animal started showing symptoms of leprosy in 1994 [31]. A sample of skin biopsy tissue from this animal had been stored using the FFPE method since 1994, from which DNA was extracted for the purposes of this study. The average length of mapped reads for sample CM1 were 80 bp, as compared to 100 bp for sample Ch4 (Table 1). The reduction in average fragment length for sample CM1 is likely due to the FFPE preservation, which is known to cause fragmentation of DNA [63], as well as the greater time duration for which this sample was stored (20 years) as compared to sample Ch4 (nine years).

Cynomolgus macaques, also known as crab-eating or long-tailed macaques, cover a broad geographic distribution in southeast Asia and have had a long history of contact with human populations [64]. These macaques have been found to be infected with pathogens such as cercopithecine herpesvirus 1 [65], simian foamy viruses [66,67], the MTBC [68], and *Plasmodium* species [69]. In the case of MTBC infection, prevalence is higher in macaques from Thailand, Indonesia, and Nepal, where tuberculosis is endemic, and lower in Gibraltar and Singapore, where tuberculosis is not endemic [68]. The Philippines ranks first in the Western Pacific Region in terms of absolute number of leprosy cases, with about 2,000 new leprosy cases reported annually [1]. Our data suggest that *M*. *leprae* strains, such as the CM1 strain, may be transmitted between humans and nonhuman primates in countries where leprosy is endemic.

Across the three nonhuman primate *M*. *leprae* strains, the highest number of SNPs were found in the *ML0411* gene, which is known to be the most polymorphic gene in *M*. *leprae* [15]. This gene codes for a serine-rich protein and is thought to have diversified under selective pressure imparted by the human immune system [70].

According to the dating analysis, the MRCA of all *M*. *leprae* strains was estimated to exist about 3,590 YBP (95% HPD 2,808–4,606 YBP), which is congruent with a previous estimate of 3,483 YBP (95% 2,401–4,788 YBP) [19] as well as with the oldest skeletal evidence for leprosy which dates to 2,000 BCE India [71]. Our estimated *M*. *leprae* substitution rate was 6.95 × 10^−9^ substitutions per site per year, which is also similar to previous estimates [15,19].

### Screening of wild nonhuman primates for presence of mycobacterial pathogens

To assess whether mycobacterial pathogens are transmitted between humans and nonhuman primates in tuberculosis- and leprosy-endemic regions, broad phylogeographic screenings of nonhuman primate populations need to be conducted. The ring-tailed lemur populations screened in this study were not necessarily expected to show prevalence of *M*. *leprae* infection, since successful experimental or natural transmission of *M*. *leprae* has not been reported in any lemur species. Madagascar reports approximately 1,500 new leprosy cases [1] and 29,000 new tuberculosis cases [72] each year. Furthermore, a “leper colony” exists in the village of Tongobory, approximately 50 kilometers from BMSR, and interactions between the lemur populations and the surrounding local human populations [73] could lead to anthroponotic transmission of *M*. *leprae* and other pathogens. However, the lemurs included in this study did not show evidence of infection by *M*. *leprae* or members of the MTBC. In future, assessing the presence of these pathogens in lemur populations closer to Tongobory would be beneficial.

Additionally, chimpanzee populations at Ngogo, Kibale National Park, in Uganda were also screened for the presence of these mycobacterial pathogens. Uganda reports about 43,000 new tuberculosis cases [72] as well as approximately 250 new leprosy cases [1] annually. The ease of transmission of MTBC strains between different mammalian hosts underlies the need for screening wildlife for the presence of MTBC infection especially in tuberculosis-endemic regions. However, the chimpanzees screened in this study did not test positive for MTBC or *M*. *leprae* infection.

### Transmission of *M*. *leprae* between humans and nonhuman primates

The results of this study support a scenario in which human *M*. *leprae* strains were transmitted to a nonhuman primate species and have been circulating in nonhuman primates. In Africa, interactions of humans and nonhuman primates—such as through zoos or sanctuaries; via hunting for bushmeat; or due to the use of nonhuman primates for exportation, sport, entertainment, and as family pets—are major sources of pathogen transmission. In the context of mycobacterial pathogens, tuberculosis infections have been reported among wild nonhuman primates in Africa. For example, wild olive baboons are reported to have acquired tuberculosis by foraging from a garbage dump (containing infected meat products) at a tourist lodge in Kenya [17,18]. Such MTBC strains which are transmitted from humans to nonhuman primates might be circulated thereafter among different nonhuman primate species leading to the development of novel MTBC lineages. Our data suggest that the African nonhuman primates studied here acquired *M*. *leprae* either directly or indirectly from human sources. While the possibility of nonhuman primates having introduced *M*. *leprae* Branch 4 strains to humans cannot be ruled out by the phylogenetic data, genomic data from other naturally infected nonhuman primates might help answer these questions. Moreover, due to the paucity of *M*. *leprae* Branch 4 genomes, we cannot rule out the possibility that the Ch4-SM1 *M*. *leprae* sublineage is currently present in humans in West Africa and is not specific to nonhuman primates. Future studies can assess the prevalence of this sublineage in human patients as well as in other wild nonhuman primate populations from West Africa using a SNP-genotyping approach for the unique SNPs found in the nonhuman primate *M*. *leprae* strains (S2 Table).

In Asia, the geographic range of nonhuman primates overlaps with human settlements, and contact between the two has increased due to human encroachment upon their habitats, hunting, and trapping activities. There is a high demand for nonhuman primates such as macaques in biomedical research, pet trade, as performing animals, and as food [74]. Due to their religious significance in Hinduism and Buddhism, macaques are respected in most of Southeast Asia and are often included in religious festivities of the local populations. They are also a prominent species in monkey temples, which serve as popular tourist attractions. These temple settings provide ample opportunities for physical contact due to tourists feeding the monkeys as well as the monkeys climbing on, biting, and scratching tourists [66]. Such interactions significantly increase the risk for pathogen transmission between humans and macaques.

The absence of leprosy cases in the wild nonhuman primate populations screened in this study is suggestive of the rarity of naturally occurring leprosy in nonhuman primates. However, naturally occurring leprosy has been reported primarily from nonhuman primates from West Africa and the Philippines, whereas the populations we screened were from Uganda and Madagascar, which is a limitation of our study.

### Summary

To the best of our knowledge, this is the first paper to report the genomes of nonhuman primate *M*. *leprae* strains. While previous studies have shown that *M*. *leprae* strains can be transmitted to nonhuman primates, we did not know if naturally occurring leprosy in nonhuman primate was due to incidental infections by human *M*. *leprae* strains or by *M*. *leprae* strains specific to nonhuman primates. Our results suggest that nonhuman primates, such as chimpanzees and sooty mangabeys in Africa and cynomolgus macaques in Asia, may acquire *M*. *leprae* strains from humans as well as transmit these strains between themselves. The wild nonhuman primate populations from Madagascar and Uganda screened in this study tested negative for the presence of mycobacterial infection. Further phylogeographic screenings of nonhuman primates in leprosy-endemic countries are necessary, because the prevalence of leprosy-causing bacteria in nonhuman primate populations could have important implications for leprosy control and primate conservation strategies.

## Supporting information

S1 Table Case details of the three nonhuman primates with leprosy included in this study. (XLSX)

S2 Table Summary of SNPs found in the nonhuman primate *M*. *leprae* strains. (XLSX)

S1 File List of positions in the *M*. *leprae* reference genome excluded from phylogenetic analyses. (TXT)

S1 Fig Maximum parsimony tree of *M*. *leprae* strains. *M*. *lepromatosis* was used as an outgroup to root the tree (branch truncated for clarity). Bootstrap support estimated from 1,000 replicates is given next to each internal branch. The five *M*. *leprae* branches are highlighted. The nonhuman primate *M*. *leprae* genomes sequenced in this study are marked in red.
(TIFF)

S2 Fig Neighbor joining tree of *M*. *leprae* strains. *M*. *lepromatosis* was used as an outgroup to root the tree (branch truncated for clarity). Bootstrap support estimated from 1,000 replicates is given next to each internal branch. The five *M*. *leprae* branches are highlighted. The nonhuman primate *M*. *leprae* genomes sequenced in this study are denoted in red.
(TIFF)

S3 Fig Map showing the geographic ranges of chimpanzees (Red) and sooty mangabeys (Blue) in Africa. The overlap between the two species’ ranges is shown in purple. The map was generated using RStudio.
(TIFF)
