# Supplementary figures and images for: Mycobacterium leprae genomes from naturally infected nonhuman primates
Source: PLoS Negl Trop Dis. 2018 Jan 30;12(1):e0006190. doi: 10.1371/journal.pntd.0006190 (PMC5790234; doi:10.1371/journal.pntd.0006190)

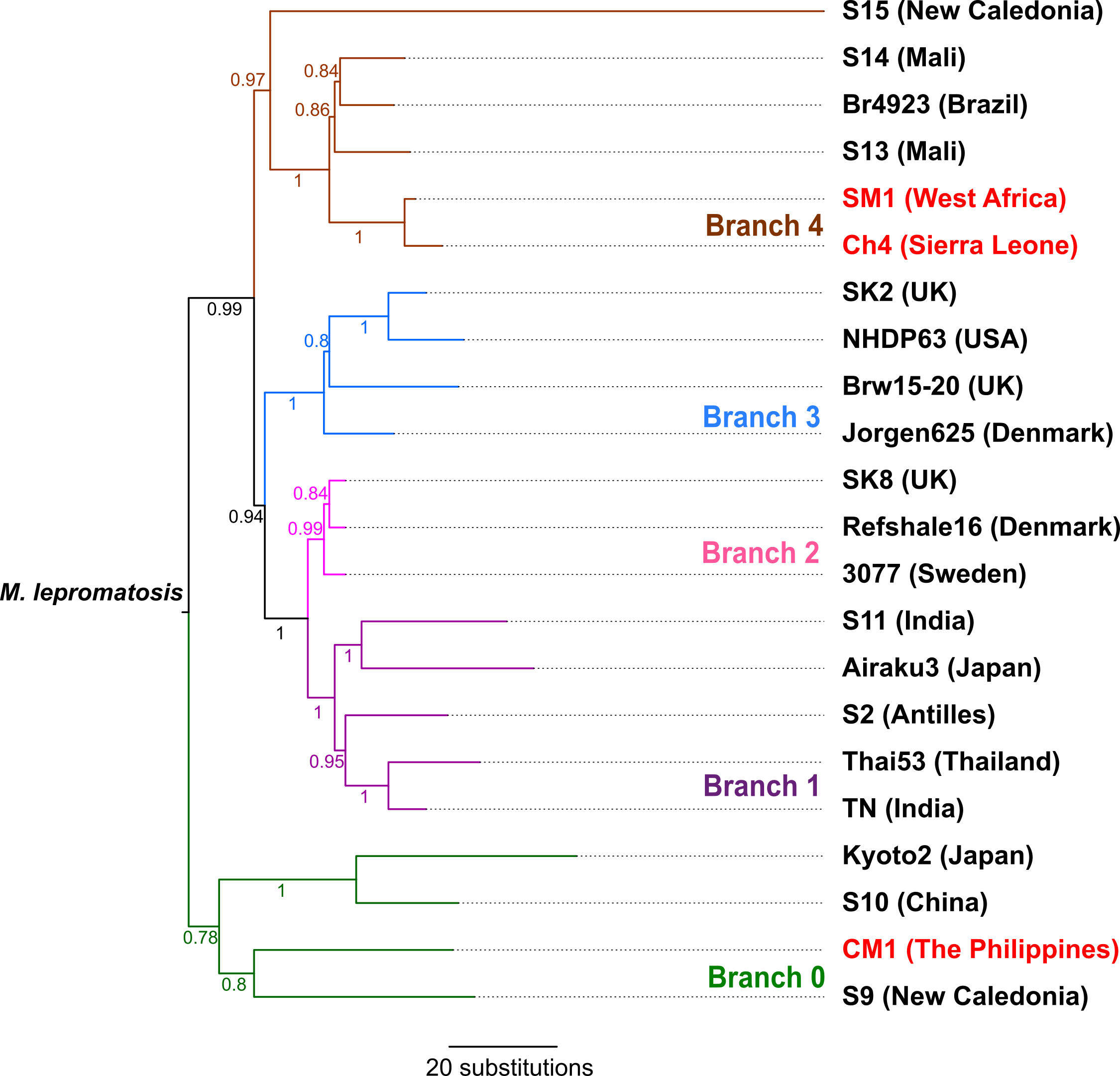

Supplement: S1 Fig — M. lepromatosis was used as an outgroup to root the tree (branch truncated for clarity). Bootstrap support estimated from 1,000 replicates is given next to each internal branch. The five M. leprae branches are highlighted. The nonhuman primate M. leprae genomes sequenced in this study are marked in red. (TIFF) [file pntd.0006190.s004.tiff]

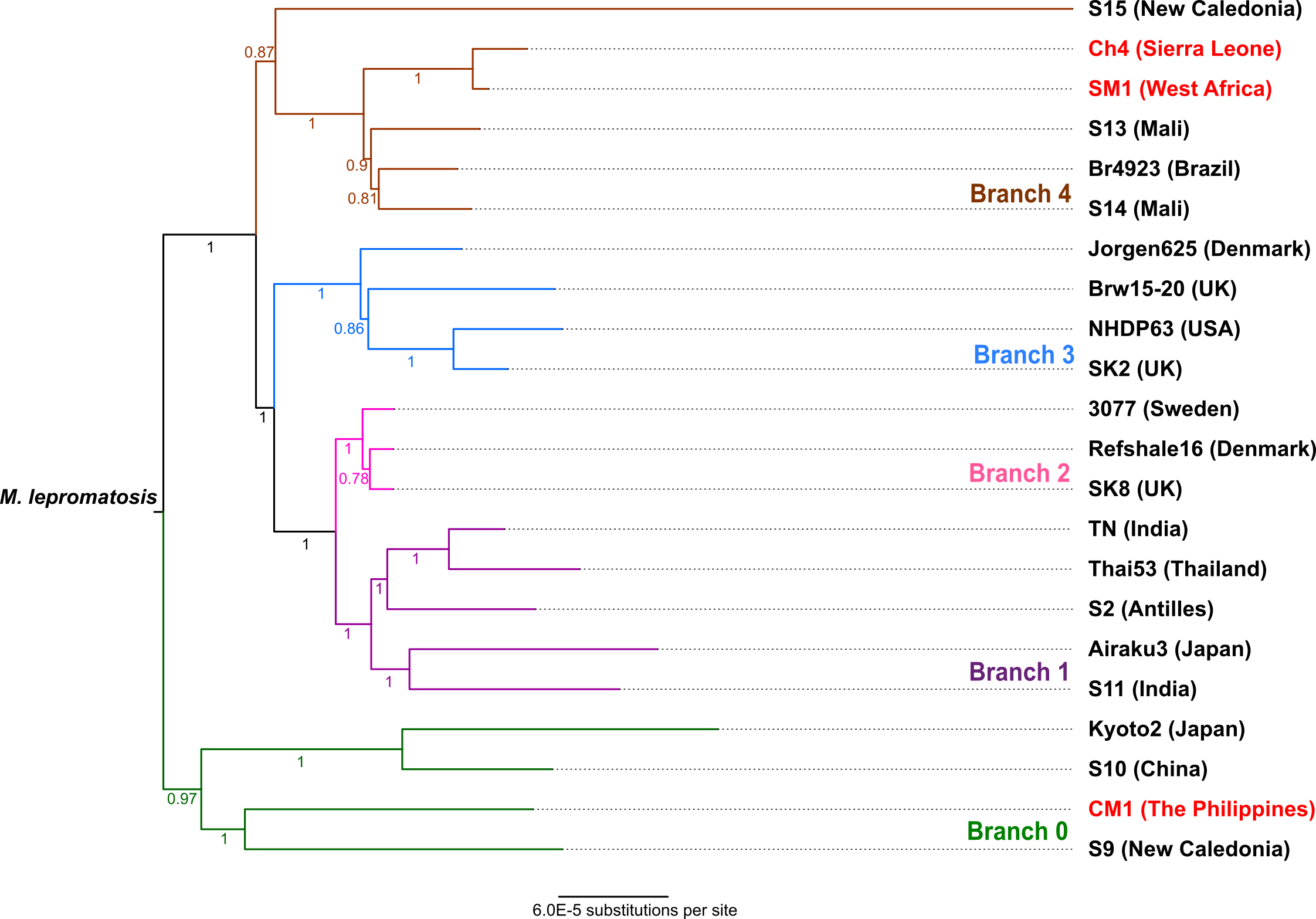

Supplement: S2 Fig — M. lepromatosis was used as an outgroup to root the tree (branch truncated for clarity). Bootstrap support estimated from 1,000 replicates is given next to each internal branch. The five M. leprae branches are highlighted. The nonhuman primate M. leprae genomes sequenced in this study are denoted in red. (TIFF) [file pntd.0006190.s005.tiff]

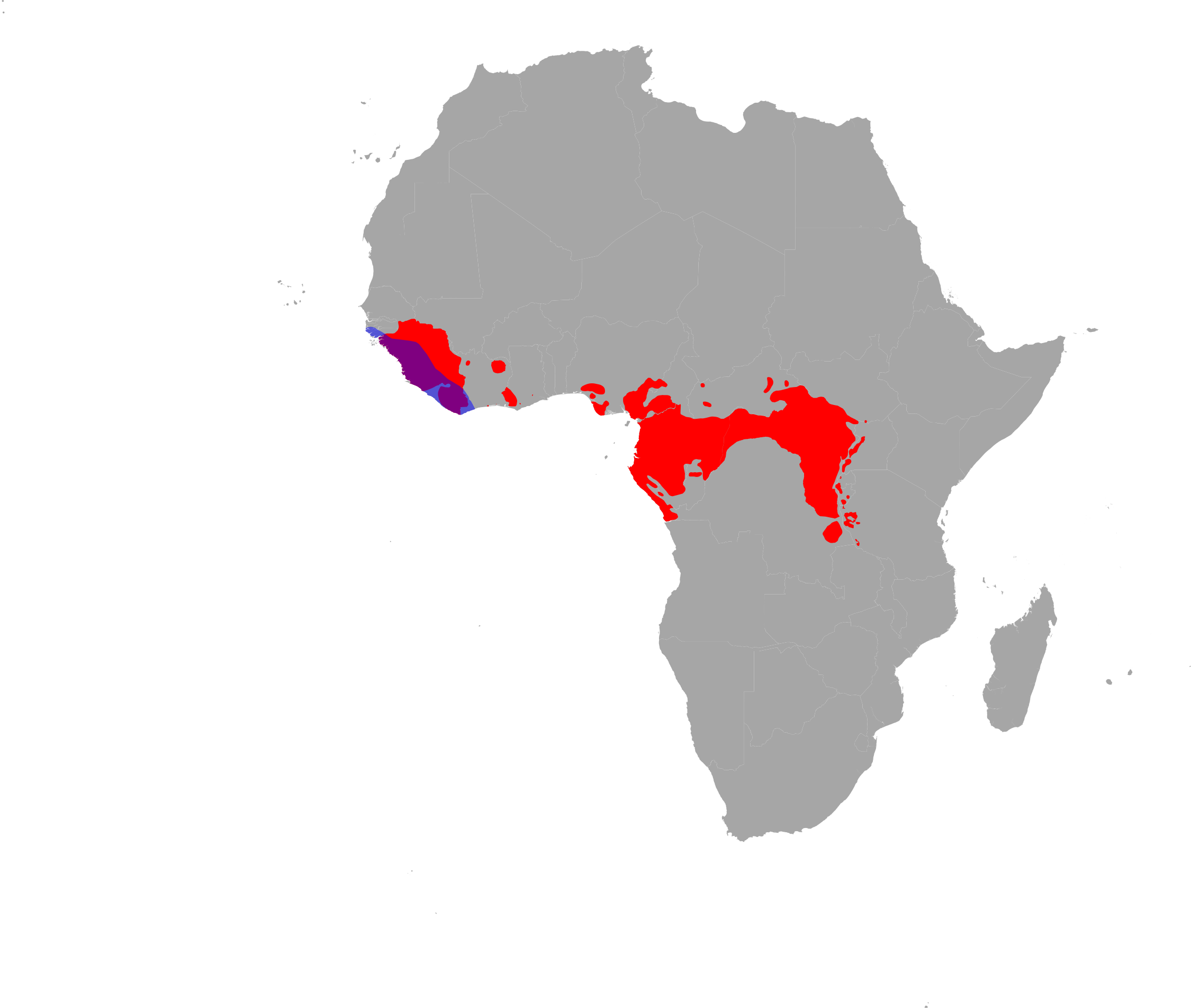

Supplement: S3 Fig — The overlap between the two species’ ranges is shown in purple. The map was generated using RStudio. (TIFF) [file pntd.0006190.s006.tiff]
